# Heatwaves are detrimental to fertility in the viviparous tsetse fly

**DOI:** 10.1098/rspb.2023.2710

**Published:** 2024-03-13

**Authors:** Hester Weaving, John S. Terblanche, Sinead English

**Affiliations:** ^1^ School of Biological Sciences, University of Bristol, Bristol, UK; ^2^ Department of Conservation Ecology & Entomology, Stellenbosch University, Stellenbosch, South Africa

**Keywords:** heatwave, lethal limits, sublethal effects, thermal fertility limits, thermal sensitivity of fertility, reproduction

## Abstract

Heatwaves are increasing in frequency and intensity due to climate change, pushing animals beyond physiological limits. While most studies focus on survival limits, sublethal effects on fertility tend to occur below lethal thresholds, and consequently can be as important for population viability. Typically, male fertility is more heat-sensitive than female fertility, yet direct comparisons are limited. Here, we measured the effect of experimental heatwaves on tsetse flies, *Glossina pallidipes*, disease vectors and unusual live-bearing insects of sub-Saharan Africa. We exposed males or females to a 3-day heatwave peaking at 36, 38 or 40°C for 2 h, and a 25°C control, monitoring mortality and reproduction over six weeks. For a heatwave peaking at 40°C, mortality was 100%, while a 38°C peak resulted in only 8% acute mortality. Females exposed to the 38°C heatwave experienced a one-week delay in producing offspring, whereas no such delay occurred in males. Over six weeks, heatwaves resulted in equivalent fertility loss in both sexes. Combined with mortality, this lead to a 10% population decline over six weeks compared to the control. Furthermore, parental heatwave exposure gave rise to a female-biased offspring sex ratio. Ultimately, thermal limits of both survival and fertility should be considered when assessing climate change vulnerability.

## 1. Introduction

Heatwaves, defined as at least 3 consecutive days with maximum temperatures above the daily 90th percentile [1], are becoming more frequent, prolonged and intense due to climate change [2–4]. Extreme temperatures are likely to have a greater impact than rising mean temperatures alone as they force animals towards or beyond physiological limits [5,6]. For many species, these limits are already being regularly surpassed [7,8], which can result in mass mortality events. For example, a single day of Australian heatwave caused the death of 45 000 flying foxes [9,10], and in Europe, a 2003 heatwave caused an excess of 70 000 human mortalities [11]. Forecasting which species are most vulnerable to extreme temperature events can help predict further biodiversity losses and geographic range shifts [12,13].

Species’ thermal vulnerability can be estimated using a variety of indices such as warming tolerance and thermal safety margins. These terms are used interchangeably to describe the average environmental temperature rise an animal can cope with before death, and the difference between a species' upper thermal limits and experienced maximum environmental temperatures [14–17]. Two metrics widely used to estimate upper thermal limits are the critical thermal maximum, or CT_max_, which is the maximum temperature at which biological function is lost (e.g. loss of coordinated movement, response to stimulus [18]), and lethal temperature, where LT80 indicates the temperature at which there is 80% mortality in a population [19]. However, recently the utility and accuracy of these indices have come into question due to the lack of incorporation of factors such as evolutionary adaptation, acclimation capacity, and thermoregulatory behaviours [17]. Additionally, these metrics do not consider sublethal effects on major fitness components such as reproduction and growth. Damage to these traits often occurs at less extreme temperatures than those which are lethal to animals [20,21], so such sublethal effects can potentially have more prevalent and sustained impact on populations than lethal temperatures [17]. For example, even small changes to female fertility can determine population growth, and therefore persistence [22]. For this reason, a multiple trait-based approach has been suggested to estimate species vulnerability that allows for factors such as adaptation and includes sublethal limits for more robust estimates [17,23,24].

The temperature at which an animal becomes infertile tends to occur well below upper thermal limits [20,21]. Studies show that high temperature affects sperm count, viability and motility, oocyte development and maturation, and causes damage to DNA, RNA and protein synthesis [25–27]. Damage to male fertility typically occurs at lower temperatures than for females [20,28,29], likely due to a requirement of low temperature for spermatogenesis [30]. As a result, a population may become inviable due to infertility even if upper thermal limits are not reached, meaning that vulnerability indices such as warming tolerance and thermal safety margins could underestimate the effects of climate change on population dynamics [23]. A recent study on 43 *Drosophila* species found that, for around half of species, infertility occurs below lethal limits and, moreover, that temperatures which define male infertility better match species distributions than LT80 or CT_max_ [31]. The study also found that CT_max_ and fertility measures are not well correlated, indicating that it will be necessary to estimate fertility limits experimentally even for those species where CT_max_ is known.

Fertility thermal tolerance studies use a variety of methodologies and trait measurements [23], making it difficult to compare species and incorporate fertility estimates into vulnerability indices. In contrast, upper thermal limits are better defined (although see e.g. [32,33]), so can be used in large-scale global analyses to identify traits that make species most vulnerable to climate change [34–36]. Therefore, two standard metrics of fertility have been proposed: first, the thermal fertility limit [23], which is a binary measure of fertility, considering individuals as fertile or infertile, measuring the temperature and duration of thermal exposure at which viable offspring production is prevented. The second metric is the thermal sensitivity of fertility, which captures the number of viable offspring produced at a given temperature [37]. Within this framework, there are further methodological considerations. Thermal exposure can be static or variable, with the former easily repeatable and useful for calculating thermal tolerance curves, but the latter potentially more ecologically relevant. Life stages also vary in sensitivity, for example in the flour beetle, *Tribolium castaneum*, heat stress in the larval stage results in permanent adult male sterility, but there is no fertility change if pupae are exposed to heat stress [20,38]. Unlike CT_max_, which typically results in insect mortality, fertility can recover [39]. Therefore, monitoring animals over a suitable timeframe offers insights into population viability. Finally, thermal fertility limit data are largely limited to model organisms, and there are few studies that compare male and female fertility directly (but see [20,37,40–42]). Therefore, researchers are calling for measurements on a wider variety of species where heat-treated sexes are measured independently under similar conditions [21,43].

Here, we investigate the effect of a 3-day heatwave on mortality and reproductive output of *Glossina pallidipes*, a disease vector of animal African trypanosomiasis in sub-Saharan Africa [44]. We expose either males or females to simulated experimental heatwaves designed to mimic a daily fluctuation in maximum, but not minimum, ambient temperature. Tsetse are unusual insects in that they are viviparous: females are live bearing, producing a single larva at a time which feeds *in utero* upon a milk-like secretion [45]. Upon larviposition, the offspring is a similar size to the adult female, representing substantial maternal investment [46]. Tsetse flies only produce a maximum of approximately 10 offspring during their lifetime meaning that population persistence is vulnerable to any increases in mortality or changes to reproductive output [47], making them an interesting study for thermal limits. Our study had three main aims: first, to determine if thermal fertility limits occur at temperatures below lethal temperatures in tsetse; second, to ascertain any differences between male and female thermal fertility limits and sensitivity, and monitor recovery time; and, third, to investigate how parental thermal stress affects offspring fitness by measuring offspring mass, sex ratio and CT_max_. We predicted that thermal fertility limits would occur at temperatures below lethal limits, but in contradiction to studies from other animals, that female fertility would be more vulnerable to heat than male fertility due the unusually high reproductive investment observed in female tsetse.

## 2. Methods

### (a) Colony conditions

Batches of approximately 900 *G. pallidipes* pupae were ordered from the International Atomic Energy Agency (IAEA) in August 2022 and December 2022, for replicate one and two respectively. IAEA colony conditions are 24–25°C and 75–80% relative humidity (RH) for adult tsetse and 25°C and 75% RH for pupae [48]. Pupae were kept in a climate-controlled room at 25°C and 80% RH. Upon emergence, adults were briefly chilled using a chest fridge (maintained between 6 and 2°C using a RS Pro Dual Datalogger with T type thermocouples) and sorted daily into single sex cages consisting of no more than 10 individuals. Daily collection ensured flies were virgins and any deformed or undersized individuals were discarded.

Flies were fed 24 h post cold exposure and then three times weekly on sterile, defibrinated horse blood (TCS Biosciences, Buckingham, UK), containing 200 µl of ATP (adenosine 5′-triphosphate disodium) per 25 ml as a feeding stimulant. Blood was covered with a silicon membrane and heated to 36°C (Flexible Heated Hoses, Birmingham, UK).

### (b) Heatwave treatment

**Figure 1.**
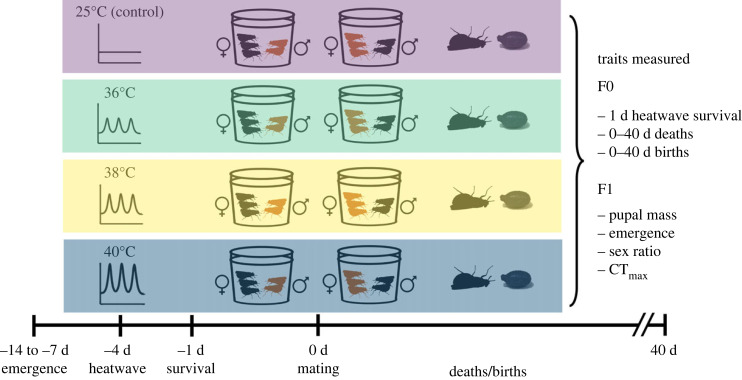
Experimental overview. Temperatures indicate heatwave maximums during water-bath exposure. Coloured tsetse silhouettes show that either male (♂) or female (♀) tsetse have been exposed to heatwave. Timeline is given in d days. Deaths and births of F0 flies were taken three times weekly.

For each trial, approximately 200 flies underwent a thermal treatment of 25 (control), 36, 38 or 40°C for 2 h for 3 consecutive days to mimic heatwave conditions (figure 1). The remaining flies underwent no thermal treatment but were walked around the laboratory as a handling control. Emergence dates were split between treatment groups as evenly as possible. These thermal treatments were chosen because a heatwave is defined as a minimum of 3 consecutive days with maximum temperatures above the daily 90th percentile temperature [1], and *G. pallidipes* CT_max_ is around 43°C [33,49]. Air temperatures within and above this range are regularly experienced in the field, see e.g. [50] at Rekomechi Research Station, Zimbabwe.

Treatments occurred between 11.00 and 15.00 in Grant LTC4 refrigerated circulating liquid baths containing RO water with TX150 heating circulators (Grant Instruments, Cambridge). Only two water baths were available for the four temperature treatments, so they were alternated daily between morning and afternoon to minimize any potential diurnal effects. Cages were placed in zip lock bags so they could be submerged in the water baths. Flies were returned to standard colony conditions at 25°C once the treatment had finished. iButtons were used to record temperatures of the water baths at a sampling rate of every 30 s, for which data can be found in the electronic supplementary material, Table S1. All heatwave temperatures were within 0.5°C of the target set temperature, except the 40°C temperature during trial 1, where on day 3, mean temperature + s.d. was 37.14 + 2.54°C, therefore data for these flies were excluded from the analysis.

### (c) Sex mixing

As one of our aims was to determine the difference in thermal sensitivity of fertility between males and females, we mixed treated flies with untreated individuals of the opposite sex. As females emerge first, they were approximately 1 day older than males (electronic supplementary material, table S2) at the time of mixing. Males become sexually mature by one week old, so all males were a minimum of one week old at the time they were mixed with females, to ensure both sexes were sexually mature [51]. We mixed three female and two male flies per cage the morning after the last day of heatwave using the chest fridge described above. This ratio was selected as female longevity is reduced in equal sex ratio cages due to male harassment [52]. Cages were made from modified 150 ml cylindrical containers (70 × 73 mm) with mesh fabric (2.5 mm) on the top and bottom (electronic supplementary material, figure S1). Each container was placed on top of a pot to catch deposited pupae which are small enough to fall through the mesh in their larval form.

### (d) Thermal lethal limits

Dead flies were counted 24 h after the heatwave treatment had finished. Thereafter, once flies were put into mixed sex cages, we scored mortality in each cage three times a week (Monday, Wednesday, Friday) for six weeks after the sexes were mixed on day 0 (figure 1). Dead individuals were removed from cages and frozen at −20°C.

### (e) Thermal fertility limits and sensitivity of fertility

Pupal production was counted three times weekly to establish date of first deposition and pupal production per cage. Pupae were removed from the pots and housed in 50 ml conical tubes, each with a hole drilled into the middle of the lid for gas exchange. Each tube contained all pupae produced per cage for each week of the experiment.

### (f) Intergenerational effects

Abortions were recorded when females deposited under-sized larvae, at the first or second larval stage [53]. These pupae were stored in 70% ethanol. Once a week, F1 pupae were weighed using an Ohaus Explorer EX124 balance (Ohaus Europe, Switzerland; accurate to 0.001 mg). A final tally of F1 emergence success and sex ratio was taken six weeks after the last pupa was deposited, as at 25°C pupation lasts approximately one month [54].

A random selection of F1 flies were chosen (*n*∼20 per treatment group and sex) for CT_max_ measurement. CT_max_ assays were run using the two water baths described previously, connected to Perspex organ pipes which were pumped with water. Grant Labwise software (version 2.1.2, Grant Instruments, Cambridge, UK) was used to set a program of 10 min acclimation at 25°C followed by ramping at a rate of +0.1°C min^−1^, which is considered ecologically relevant from temperature data [33]. Two thermocouples (Type T) and a digital thermometer monitored temperature in empty pipes which is equivalent to body temperature due to the small body size of tsetse flies [33].

Flies were transferred into pipes by knocking them down using 8 μl of sevoflurane anaesthetic pipetted into the 50 ml conical tube through the drilled hole. Previous experiments have shown that sevoflurane does not affect tsetse mortality up to one week after admission [49], and is known to have limited off-target effects in *Drosophila* [55]. The hole was covered with cotton wool until flies were knocked down (around 2 min). Once all flies were standing the program commenced. CT_max_ trials were run by one researcher (HW). Programs on the two baths began in 20–30-minute tandem so a maximum of 10 flies were assessed at once. CT_max_ was defined as the temperature at which the fly stopped responding to experimenter-induced disturbance of the organ pipes.

### (g) Statistics

All data analysis was completed in R Studio (v. 3.5.1; [56]). Models were verified by examining residuals for normality (where appropriate), heteroscedasticity and outliers, as well as model deviance and collinearity. For each model, we assessed the effect of the interaction between treatment temperature (25, 36, 38 or 40°C) and the sex exposed (male heatwave or female heatwave) and controlled for differences between trials (∼ temperature * [exposed sex] + trial). We present all results as unstandardized effect sizes [57], and full model results in the electronic supplementary material, tables S3 to S12.

### (h) Thermal lethal limits

The difference in proportion survival per cage for each treatment group 24 h post heatwave was calculated using a generalized linear model with binomial distribution with ‘logit’ link. As 100% of individuals died in the 40°C treatment the brglm2 (v. 0.9 [58]) package was used to reduce bias due to complete separation in the model [59].

A Cox proportional hazards model was used to test how heatwave affected longevity over the six weeks of the experiment. We used packages ‘survival’ (v. 3.2.13; [60]) and ‘coxme’ (v. 2.2-18.1 [61]) to fit and visualize the data. No flies survived a 40°C heatwave so they were not included in the analysis. Only flies that experienced the heatwave were included in the model (*N* = 117, 124, 125 for 25, 36 and 38°C groups, respectively). Individuals that were still alive by the end of the experiment were censored. Cage code was used as a random factor to account for variation between cages (148 cages).

### (i) Thermal fertility limits and sensitivity of fertility

The time in days until the production of the first pupa per cage was modelled as a mixed effect Cox proportional hazard survival analysis, to assess if there was a delay to the production of offspring due to heatwave. To assess the effect of heatwave on fertility while accounting for female mortality, we completed a time-step analysis where, for each week, the number of females successfully producing pupae was the response variable and the log of the number of living females at that time point was included as an offset (as in [62]). As 52% of the observations were zero values (388/740), we used a zero inflated model with Poisson distribution using the ‘glmmTMB’ package (version 0.8-8; [63]), with a single zero inflation parameter applied to all observations. We included cage code as a random factor, allowing for different slopes between cages over weeks, to account the auto-correlation due to repeated measures of the same cage [64].

To investigate population viability, we measured the effect of treatment on total fly population over time in a general linear model with Gaussian distribution and an ‘identity’ link function. We calculated the total fly population as [births + total living individuals] per measurement day per cage. We only included births where offspring later successfully emerged. We investigated the three-way interaction between heatwave temperature, exposed sex and time on the total population, with cage code as a random factor.

### (j) Intergenerational effects

A series of generalized linear models were run to assess the difference between treatment groups in offspring mass, emergence success, sex ratio and CT_max_. The difference in pupal mass between groups was assessed by a general linear model with Gaussian distribution and ‘identity’ function. Cage code was used as a random factor. The proportion of successfully emerged offspring and their sex ratio were calculated per cage. Generalized linear models with binomial distribution and ‘logit’ link were used for analysis. Due to model overfitting, the random factor of cage code was not included in either the sex ratio model or emergence success model. Finally, the effect of parent heatwave treatment on offspring CT_max_ was assessed by a general linear model with Gaussian distribution and ‘identity’ link function. We included pupal mass as a factor, as size can affect CT_max_ estimates in tsetse [49].

## 3. Results

### (a) Thermal lethal limits

**Figure 2.**
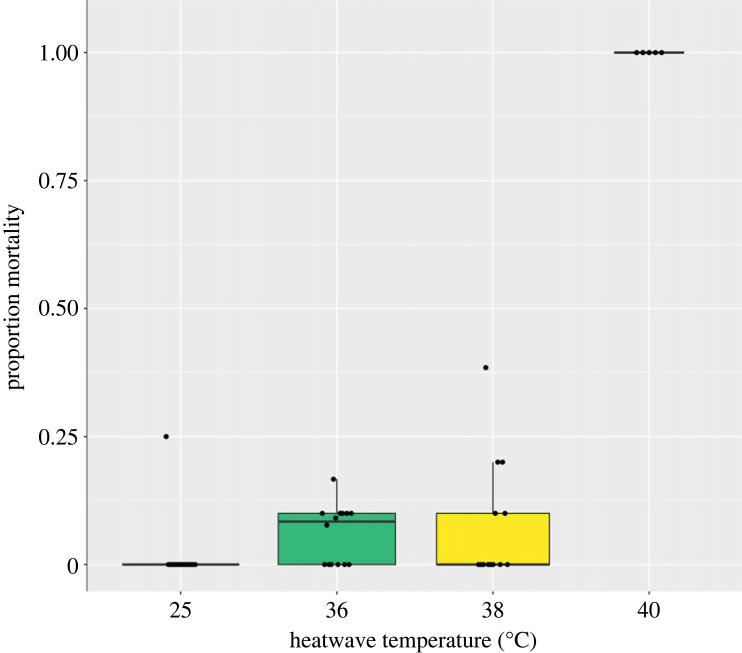
Proportion mortality of adult tsetse (*G. pallidipes*) 24 h following heatwave exposure. The median, interquartile range and confidence interval (boxes and lines) as well as the individual data (black points) are shown. Proportion survival was taken per cage, left to right: *n* = 14, 14, 13, 5. Each cage contained approximately 10 flies. *N* is lower in the 40°C group due to failure of the water bath to reach a suitable temperature in trial 1.

A 3-day heatwave peaking at 38°C caused 8% direct mortality in flies 24 h after exposure (figure 2; odds ratio (95% CI) = 1.12 (0.25–5.07); *z* = 0.14; *p* = 0.89). Mortality sharply increased to 100% when the heatwave peaked at 40°C (odds ratio (95% CI) = 1497 (70–31 987); *z* = 4.68; *p* < 0.001). There was no difference between male and female mortality (odds ratio (95% CI) = 0.19 (0.01–3.70); *z* = −1.10; *p* = 0.27), and no interaction between sex and temperature on mortality (electronic supplementary material, table S3). There was no difference between trials (odds ratio (95% CI) = 0.95 (0.42–2.13); *z* = −0.13; *p* = 0.90).

**Figure 3.**
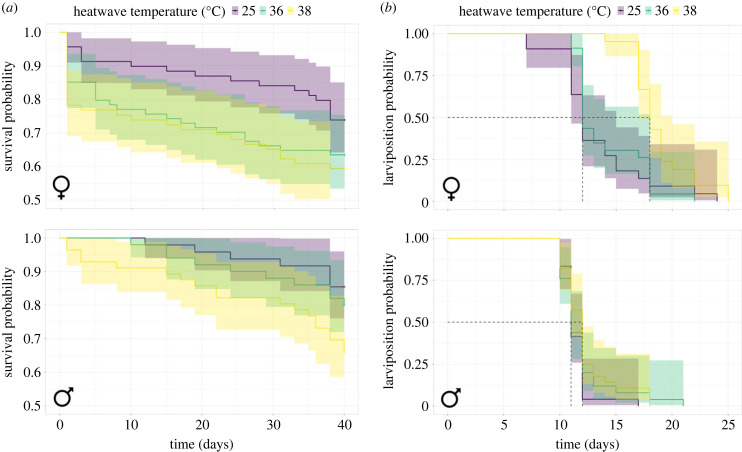
Cox survival analyses for (*a*) probability of survival for adult tsetse (*G. pallidipes*) over six weeks after either female (♀) or male (♂) adult flies were exposed to heatwave (*n* = 212 females, 154 males). Crosses indicate censored individuals which survived until the end of the experiment. (*b*) Probability of first larviposition event for adult tsetse after heatwave (*n* = 143 cages). Ninety-five per cent confidence intervals are shaded.

Survival was assessed over the six-week period following the heatwave by Cox proportional hazards model. The largest effect in the model was trial, with more flies dying in the second trial than the first (HR (95% CI) = 2.74 (1.73–4.35); *z* = 4.28; *p* < 0.001). Temperature had less of an effect in the model, with potentially greater numbers of flies dying in the 38°C group (HR (95% CI) = 1.89 (0.92–3.90); *z* = 1.72; *p* = 0.09) and the 36°C group (figure 3*a*; HR (95% CI) = 1.77 (0.86–3.65); *z* = 1.55; *p* = 0.12), but high uncertainty due to wide confidence intervals. Males were potentially at less risk of death than females (HR (95% CI) = 0.48 (0.18–1.28); *z* = −1.46; *p* = 0.14), but again this result is uncertain due to wide confidence intervals. There was no interaction between exposed sex and heatwave temperature, indicating that any effect of heatwave temperature on mortality over the six-week experiment did not depend on which sex was exposed (electronic supplementary material, table S4).

### (b) Thermal fertility limits and sensitivity of fertility

We investigated the effect of heatwave on the number of days until the first larva was produced in each cage. Heatwave temperature had a large effect on the number of days until the first larviposition event, with a greater delay when flies were exposed to 38°C compared to the control group (figure 3*b*; HR (95% CI) = 0.24 (0.11–0.50); *z* = −3.74; *p* < 0.001). Whether males or females were heatwave-exposed also had a large effect; females that were heat-exposed took longer to produce their first pupae than untreated females paired with heatwave-exposed males (HR (95% CI) = 2.88 (1.38–5.99); *z* = 2.83; *p* = 0.005). Note that these data include 25°C control groups, indicating a negative handling effect on females. However, a potential interaction between heatwave temperature and sex exposed indicates that this difference could be due to a greater effect of heatwave on females at 38°C (electronic supplementary material, table S5, S6; HR (95% CI) = 2.61 (0.96–7.05); *z* = 1.89; *p* = 0.06). A heatwave of 38°C delayed the larviposition of the first pupa when females were exposed to heatwave, compared to the control, from a median (IQR) of 12 (11–15) to 18 (17–19) days (figure 3*b*). When males were exposed, there was no meaningful delay between the 38°C group and the control; both groups produced their first offspring at 12 (11–12) days (electronic supplementary material, table S5, S6). Overall, there was potentially a small effect of trial (HR (95% CI) = 0.68 (0.44–1.04); *z* = −1.78; *p* = 0.08).

We assessed fertility across time in a zero-inflated model using an offset of the number of living females in each cage to account for mortality during the experiment. Results are expressed as the mean difference in number of pupae produced per cage each week. The variable with the largest effect in the model was heatwave temperature. Flies that underwent a heatwave of 38°C produced fewer offspring than the control groups (mean difference ± SE = −0.65 ± 0.33; *z* = −1.99; *p* = 0.05). However, this effect was not seen when flies experienced a heatwave of 36°C (mean difference ± SE = −0.03 ± 0.28; *z* = −0.12; *p* = 0.90). The number of pupae produced marginally increased over the course of the experiment (regression coefficient ± SE = 0.11 ± 0.06; *z* = 1.95; *p* = 0.05). There was no meaningful difference between heatwave treated sexes in fertility (mean difference _(males–females)_ ± SE = 0.25 ± 0.26; *z* = 0.99; *p* = 0.32). There were no meaningful interactions between sex exposed and temperature, time and temperature, or sex exposed and time (electronic supplementary material, table S7). There was no three-way interaction between time, sex exposed, and heatwave temperature (electronic supplementary material, table S7), and no difference in fertility between trials (mean difference _(trial 2–trial 1)_ ± SE = 0.001 ± 0.06; *z* = 0.02; *p* = 0.98).

**Figure 4.**
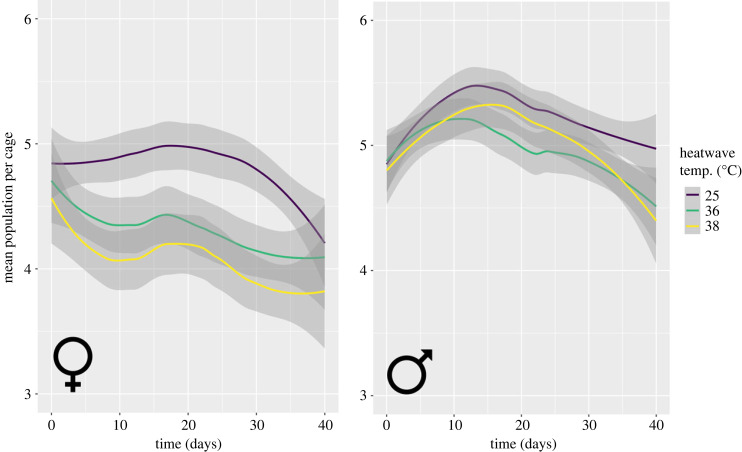
Mean tsetse (*G. pallidipes*) population per cage after female (♀) or male (♂) heatwave. The number of individuals per cage was calculated by adding the number of living flies to the number of births at each time point. Lines are drawn with geom_smooth using formula = y ∼ x and method = ‘loess’ and are displayed with 95% confidence intervals.

To assess population viability, we modelled mortality and births over time to calculate changes in the mean population per cage (figure 4). A 38°C heatwave reduced the mean number of flies per cage by 0.54 ± 0.25 (mean difference ± SE) compared to the control groups (*t* = −2.17; *p* = 0.03). A heatwave of 36°C reduced population size by 0.36 ± 0.24 compared to the control, but note uncertainty due to high error (*t* = −1.47; *p* = 0.14). Overall, population size marginally decreased over the course of the experiment (regression coefficient ± SE = 0.008 ± 0.003; *t* = −2.65; *p* = 0.01). There was some evidence that time interacted with heatwave temperature: as the experiment progressed, population growth rates decreased more in the 38°C group than the control groups (mean difference ± SE = −0.010 ± 0.004; *t* = 1.50; *p* = 0.13; electronic supplementary material, table S8). There was no interaction between sex exposed and time, or sex exposed and temperature (electronic supplementary material, table S8). There was no difference in population growth rate whether males or females were exposed to heatwave (mean difference _(males–females)_ ± SE = 0.26 ± 0.25; *t* = 1.05; *p* = 0.29). We found that a three-way interaction between time, exposed sex and heatwave temperature was of no meaningful effect (electronic supplementary material, table S8). Overall, population sizes were smaller in trial 2 than trial 1 (mean difference _(trial 2–trial 1)_ ± SE = −0.43 ± 0.13; t = −3.24; *p* = 0.001). We display mortality and fertility effects for each sex separately in electronic supplementary material, figure S3.

### (c) Intergenerational effects

**Figure 5.**
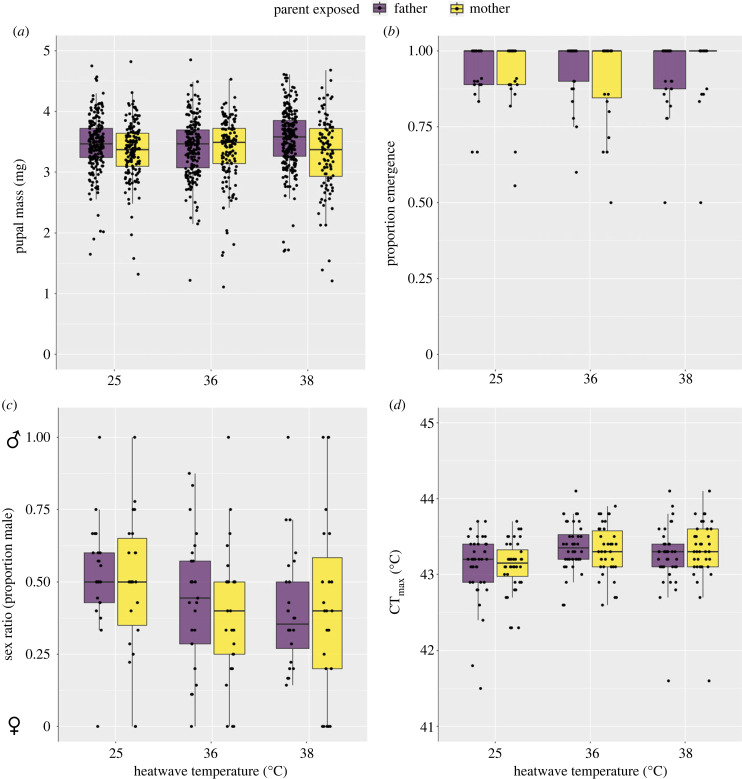
Intergenerational effects on offspring of tsetse (*G. pallidipes*) after parents (fathers or mothers) experienced a heatwave. Cages of five flies (three female and two male) were allowed six weeks to produce offspring. Pupae were weighed the week of deposition (*n* = 1049). Proportion emergence per cage (*n* = 144) and sex ratio (1 = complete male (♂) bias, 0 = complete female (♀)) were determined per cage. Critical thermal maximum (CT_max_) was measured in a random subset (*n* = 240). The median, interquartile range and confidence interval (boxes and lines) as well as the individual data (black points) are shown.

There was no effect of parent heatwave temperature on the mass of pupae at 36°C (mean difference ± SE = −0.06 ± 0.08; *t* = −0.81; *p* = 0.42) or 38°C (mean difference ± SE = 0.07 ± 0.07; *t* = 0.98; *p* = 0.33). There was also no overall difference in pupal mass depending on whether mothers or fathers were heatwave exposed (mean difference _mother−father_ ± SE = −0.11 ± 0.08; *t* = −1.34; *p* = 0.18), or an interaction between temperature and which parent was heatwave-exposed (figure 5*a*; electronic supplementary material, table S9). Pupae were lighter in trial 2 compared to trial 1 (mean difference _trial 2–trial 1_ ± SE = −0.10 ± 0.05; *t* = −2.00; *p* = 0.05). We also found no effect of parent heatwave temperature on emergence success at 36°C (odds ratio (95% CI) = 0.87 (0.36–2.05); *t* = −0.33; *p* = 0.74) or 38°C (odds ratio (95% CI) = 1.01 (0.45–2.27); *t* = 0.02; *p* = 0.98; figure 5*b*). There was no difference in emergence success depending on whether mothers or fathers were exposed (odds ratio (95% CI) = 1.70 (0.77–3.82); *t* = 1.32; *p* = 0.19), or interaction between these variables (electronic supplementary material, table S10). There was no difference between trials in offspring emergence success (odds ratio (95% CI) = 1.25 (0.75–2.09); *t* = 0.87; *p* = 0.38). At higher heatwave temperatures, there was a skew towards the production of females (figure 5*c*). More females were produced by parents that experienced a 38°C heatwave compared to the 25°C groups (odds ratio [of proportion males produced] (95% CI) = 0.56 (0.36–0.85); *z* = −2.69; *p* = 0.01), but there was no difference if parents experienced 36°C (odds ratio (95% CI) = 0.73 (0.47–1.13); *z* = −1.41; *p* = 0.16). There was no difference in sex ratio of offspring whether mothers or fathers were exposed to heatwave (odds ratio _mother−father_ (95% CI) = 1.01 (0.62–1.63); *z* = 0.03; *p* = 0.98), or interaction effect (electronic supplementary material, table S11). There was also no difference in sex ratio between trials (odds ratio _trial 2−trial 1_ (95% CI) = 0.95 (0.72 −1.26); *z* = −0.32; *p* = 0.75).

As anticipated, larger flies had greater CT_max_ (regression coefficient ± SE = 0.66 ± 0.17; *t* = 3.91; *p* < 0.001). Parental heatwave temperature resulted in marginally higher offspring CT_max_ when either parent was exposed to 36°C (figure 5*d*; mean difference ± SE = 0.21 ± 0.11; *t* = 1.86; *p* = 0.06), but this result is uncertain due to high error. There was no difference if either parent was exposed to 38°C (mean difference ± SE = 0.10 ± 0.11; *t* = 0.89; *p* = 0.37). There was no difference between mother and father heatwave on offspring CT_max_ (mean difference _mother−father_ ± SE = −0.06 ± 0.12; *t* = −0.52; *p* = 0.60), nor was there an interaction effect with temperature (electronic supplementary material, table S12). Offspring from trial 2 had marginally lower CT_max_, although the error was large (mean difference _trial 2–trial 1_ ± SE = −0.07 ± 0.04; *t* = −1.75; *p* = 0.08). Abortions were relatively rare, so the sample size was too small for formal analysis. The largest number of abortions were produced by females paired with males that underwent a 38°C heatwave (*n* = 12). Males that underwent a 36°C heatwave and control males were paired with females that went on to have five and two abortions, respectively. For heatwave treated females, the 25°C control, 36 and 38°C group had a total of seven, six and six abortions, respectively.

## 4. Discussion

Here, we exposed male and female tsetse to a simulated 3-day long heatwave peaking at 36, 38 and 40°C. Our three key results are that, first, thermal fertility limits appear to occur close to lethal limits in tsetse; a heatwave peaking at 38°C results in a week-long delay to the onset of reproduction but does not render individuals permanently sterile. Second, this delay in reproduction occurs only if females are exposed but not males, yet, overall, sexes have an equivalent thermal sensitivity of fertility in response to heatwave. Third, intergenerational effects of heatwave cause a female-biased sex ratio in offspring, but do not strongly affect other offspring traits such as emergence success, CT_max_ and mass.

The effect of heatwave on immediate mortality did not depend on which sex was exposed. Strikingly, we found a heatwave peaking at 40°C killed 100% of individuals, but only a 2°C reduction at 38°C allowed 92% survival. At 36°C, we found there was no difference in mortality from the control. Although our sample size was low for the 40°C heatwave treatment (*n* = 5 cages, with approximately 10 flies per group), a related study on *G. pallidipes* found that survival probability falls almost to zero at 1, 2 or 3 h exposures at 40°C [65]. Despite high survival in the 38°C group immediately after the heatwave, there appeared to be potentially higher mortality risk compared to the control over the six-week experiment, and females may have been at greater risk of death than males.

Fertility loss at sublethal temperatures occurs in many endothermic and ectothermic species [25–27,66], as in the present study. However, contrary to general assumptions that male fertility is more thermally sensitive than female fertility and our own counter-prediction, we found that both sexes were equally sensitive to heatwaves [20,21,23,67]. Initially, we found a stronger impact on female fertility, with onset to reproduction delayed by one week compared to the control. Yet, overall, reproductive output was equivalently reduced over the course of six weeks for heatwave-exposed males and females. The combined effect of reduced fertility and increased mortality meant that the populations exposed to a 38°C heatwave declined by 10.8% over the six week experiment when either sex was exposed to heatwave.

Generally, studies appear to find that male gametogenesis is more temperature sensitive than female gametogenesis [67], which could explain why male fertility tends to be more sensitive to heat stress than that of females. Here, we do not find such contrasting sex differences which may be due to taxonomic variation in mechanisms of gamete production. Thermal stress experienced by males can lead to atypical spermatogenesis, producing abnormal sperm form and reduced motility [68,69]. Mature sperm are less temperature sensitive than spermatogenesis and, as a result, the effect of high temperature on male fertility can be delayed [30,31,70]. In *Drosophila* this delay results in a week-long period where there is no apparent effect of heat shock on male fertility until the mature sperm stock is used up [71]. By contrast, tsetse sperm production occurs during development and ceases upon emergence, meaning that males have their entire mature sperm stock as teneral adults [72]. The stage at which spermatogenesis occurs could therefore be an important factor when assessing sex differences in thermal fertility limits. In tsetse, male fertility will likely suffer from the effects of heatwave during the pupal stage when spermatogenesis occurs. Future work should confirm this hypothesis by examining how the provision of virgin females to heatwave treated males at regular time intervals post heatwave affects male fertility in tsetse. Additionally, the effect of heatwaves experienced by pupae on subsequent male fertility of tsetse should be examined. Finally, the physiological mechanism by which heat stress affects male fertility could be investigated, for example, by using a sperm viability assay at a range of temperatures (see electronic supplementary material, figure S2 for details on our pilot work).

Tsetse female fertility may be vulnerable to high temperature due to high maternal investment in reproduction [53,73–75]. Tsetse face a trade-off between allocating energy to current reproduction or to survival to enable future reproduction [76]. Therefore, if conditions are unfavourable, an adaptive strategy for female tsetse could be to delay reproduction either through suspended ovulation or early abortion. Abortion may occur at the egg or early larval stage in response to adverse conditions to avoid high costs of producing a larva [53,73–75]. Indeed, field studies have noted a higher rate of abortions in hotter months of the year [74]. We found no meaningful difference in abortion rates between treatment groups in the present study, but we may not have detected egg-stage abortions as microscopy is necessary at this stage [53]. The link between maternal investment and sensitivity of female fertility will need to be addressed by future comparative work among invertebrates as the high maternal investment seen in tsetse is rare within this group [53,73–75]. Damage to reproductive organs may have also caused a reproductive delay. Abnormal ovaries were found in *Glossina fuscipes fuscipes* after exposure to constant 30°C [77]. Other studies have shown that infertility can be temporary in *Drosophila melanogaster* and *T. castaneum*, indicating that physiological repair can occur over time [38,78]. Periodic dissections in tsetse investigating oocyte production and release post heatwave exposure may elucidate the mechanism of this response.

Our study resonates with studies in butterflies and beetles, which find that female fertility can be equally or more thermally sensitive than male fertility [37,42]. In the seed beetle, *Callosobruchus maculatus*, Baur and colleagues found that, for both juvenile and adult heat stress, resulting female fertility was more sensitive than male fertility [37]. They also tested the effect of mating system on thermal sensitivity of fertility and found that, in a polyandrous system, effects were more pronounced, potentially due to increased male harassment. As we tested tsetse under polygamy, this could be a reason for female sensitivity in fertility. Additionally, for seed beetles, polygamy is effective at purging deleterious male mutations through stronger male sexual selection [79,80]. Through sexual selection, successful males could have greater fecundity, counteracting some of the effects of temperature. It is unknown in tsetse if male sexual selection is stronger than female sexual selection, but this is another avenue worth investigation.

Although we cannot rule out potential longer-term effects of heatwave on male fertility, our study highlights that heatwave effects on female fertility should not be overlooked. In sexually reproducing species, female reproductive output is often more important than that of males for population viability [22], especially in polyandrous species where fertile males can mate with multiple females. Indeed, tsetse population models are often based on female mortality and fertility, assuming that there will be enough males to inseminate the population [81]. These modelling studies show that small changes in mortality are more important than small changes in fertility for population persistence [82]. However, we should note that the sterile insect technique eradicated tsetse from the island of Zanzibar, where thousands of sterile males were released to control the population [83]. Similarities can be drawn between this technique and the thermal sterility of males [21].

We found intergenerational effects in response to parental heatwave on offspring sex ratio, with more females produced after flies experience a 38°C heatwave. Sex determination in tsetse might be based on the ratio of X chromosomes to autosomes as they exhibit chromosome aneuploidy (females are XX, XXY or XXXY, and males are XY, XYY or XO), similar to *Drosophila* [84]. It is unknown, however, how temperature could affect this mechanism. From an evolutionary perspective, a female-biased sex ratio may be adaptive as females tend to be larger and longer lived than males [85,86]. Therefore, producing more females may have fitness benefits in times of stress, especially as there is strong selection against small-bodied flies at hot times of the year in the field [53,87,88]. Female biased sex ratio under high temperature has also been identified in moths [89], but in this study the authors argue that more females are produced due to favourable conditions as females are more expensive to produce. We also potentially found that if either parent experienced 36°C, their offspring had slightly higher CT_max_, however this result is uncertain due to high error. This could indicate a fitness benefit of parents experiencing a brief, moderate temperature stress (intergenerational plasticity), or that those parents which survive this stressful event produce offspring with higher thermal tolerance (selection). Previous studies have found stronger effects in other insect species. One study has shown that CT_max_ of *D. melanogaster* offspring was significantly higher when both parents were reared in a variable thermal environment [90], and another study found similar results if mothers were raised at high temperature [91]. Similarly, in the peach-potato aphid, *Myzus persicae,* one generation of high temperature increased the movement threshold and heat coma [92].

Here, we investigate the response of male and female fertility to heatwave in a disease-spreading insect. We find that thermal fertility limits in tsetse are likely to occur close to temperatures that are fatal. Unlike other insects where male fertility appears more sensitive to heat, we find an equivalent effect of heatwave on male and female thermal sensitivity of fertility. Heatwaves can transcend into the offspring generation, resulting in a female-biased sex ratio, yet do not affect other offspring traits including thermal tolerance or mass. Ultimately, female fertility limits should not be overlooked, and both thermal effects on mortality and fertility should be considered when attempting to forecast effects of local weather or climate change on population dynamics.

## Data Availability

The raw data used in this study are available from the OSF database at: https://osf.io/3yxc9/. The code used to analyse data is available from the OSF database: https://osf.io/3yxc9/. The data are provided in the electronic supplementary material [93].
